# High variation in purity of consumer-level illicit fentanyl samples in Los Angeles, September 2023–April 2025

**DOI:** 10.1016/j.drugpo.2025.104977

**Published:** 2025-08-30

**Authors:** Chelsea L. Shover, Adam J. Koncsol, Morgan E. Godvin, David Goodman-Meza, Bryce Pardo, Michelle Poimboeuf, Caitlin A. Molina, Ruby Romero, Jasmine Feng, Joseph R. Friedman

**Affiliations:** a Department of General Internal Medicine and Health Services Research, University of California, Los Angeles, United States; b Kirby Institute, UNSW Sydney, Australia; c United Nations Office on Drugs and Crime, Vienna, Austria; d Department of Psychiatry, University of California, San Diego, United States

**Keywords:** Drug checking, Harm reduction, Quantitation, Illicit drug supply

## Abstract

**Background:**

The illicit manufacture of fentanyl results in product of unknown purity, contributing to overdose risk. However, data on the purity of illicitly manufactured fentanyl (IMF) in the United States typically comes from law enforcement sources and almost no information relevant to retail-level product is made available. We aim to quantify IMF purity among samples from a community-based drug checking program operating at four geographic sites in Los Angeles, California.

**Methods:**

Drug samples (*n* = 1763) were obtained from participants who also answered an anonymous survey about sample characteristics. Qualitative and quantitative analysis were conducted leveraging directly observed mass spectrometry (DART-MS) and liquid chromatography mass spectrometry (LC/MS) respectively. LC/MS quantified a panel of compounds including fentanyl and fluorofentanyl. Composite IMF purity was estimated by adding the percent mass of fentanyl and fluorofentanyl.

**Results:**

A total of 353 samples had either fentanyl, fluorofentanyl, or both quantified between September 2023 and April 2025. Median IMF purity was 5.8 %, mean 10.0 %, SD 11.1 %, range 0.1–64.9 %. Samples expected to be fentanyl (*n* = 308) had higher median purity (7.0 %) compared to those expected to be heroin (*n* = 24, median purity 1.4 %) or other drugs (*p* < 0.001). Powder samples (*n* = 318) had higher median concentration (6.9 %) compared to pills (*n* = 11, 0.7 %) or tar (*n* = 22, 1.4 %) [*p* < 0.001]. Of expected-fentanyl samples, 42.5 % (*n* = 131) had an IMF purity of <5 %, while 17.5 % (*n* = 54) had purity over 20 %.

**Conclusions:**

We found high variation in IMF purity among samples sold as fentanyl, even among samples obtained on the same day, in the same location. This volatility likely plays a role in high overdose risk, even among people with opioid tolerance, in the fentanyl era. Further research is needed to compare these findings to other locations across the United States.

## Introduction

In the United States, the overdose mortality rate far exceeds that of other nations, and has been an enduring public health problem over recent decades (Jalal et al., 2018). The overdose crisis has been described as occurring in several ‘waves’ which can be characterized by the primary drugs involved in overdose deaths during each period (Ciccarone, 2019). The current “fourth wave” of the overdose crisis is characterized by deaths involving illicitly manufactured fentanyl (IMF) mixed with psychostimulants like methamphetamine or cocaine (Ciccarone, 2021), and increasingly with other psychoactive substances (Friedman & Ciccarone, 2025; Friedman & Shover, 2023).

The variable purity of IMF has been hypothesized to be a key driver of the overdose crisis, given the much stronger potency by weight of IMF compared to other opioids, such as semi-synthetic prescription opioids, or heroin. Nevertheless, information about the purity of IMF has been limited, and mostly based on law enforcement data (Kilmer et al., 2022; Rasanen et al., 2019; Toske et al., 2023). Quantitative testing of consumer-level samples of IMF has been performed more regularly in several Canadian drug checking programs (Delaney et al., 2023; Tobias et al., 2022) but there are limited reports in the scientific literature describing fentanyl markets in the United States (Hochstatter et al., 2025; Shover et al., 2025). The purity of consumer-level samples of IMF can be expected to differ from a simple analysis of law enforcement samples, which generally include wholesale quantities that have not yet gone through all the steps of adulteration and dilution that typically occur before final retail sale (Solimini et al., 2017; Zhu et al., 2023). Furthermore, law enforcement seizures are convenience samples and may not reflect accurate trends in illegal drug markets. Understanding variation in purity and co-detected compounds is critical to responding to the evolving challenges that IMF pose to public health.

In 2023, the most recent year for which complete data are available, Los Angeles County led the United States in number of deaths involving “synthetic opioids other than methadone,” an *International Classification of Diseases 10th Edition* code widely used in epidemiological research to represent IMF and other illicitly manufactured synthetic opioids (Friedman, 2025; Friedman & Shover, 2023). Relative to other areas of the U.S., Los Angeles was late to develop an IMF market, and deaths involving fentanyl have largely involved other drugs such as psychostimulants (Friedman & Shover, 2023; Shover et al., 2020). Attributing polysubstance deaths to unexpected fentanyl exposure (e.g., using cocaine or methamphetamine contaminated or adulterated with fentanyl) versus intentional co-use (e.g., using a speedball or goofball) has been challenging. Prevention strategies for these two scenarios differ, and testing drug product samples stands to add some clarity.

A number of harm reduction approaches, techniques, and services have emerged to combat the high death toll of the overdose crisis. These include (but are not limited to) community-based naloxone distribution, safe consumption sites, and distribution of safe drug consumption supplies (Beletsky et al., 2018; Fiorentin & Logan, 2020; Zang et al., 2023). Another promising strategy can be found in community-based drug checking services, which are becoming increasingly popular in the midst of the proliferation of IMF (Bailey et al., 2023; Delaney et al., 2023). Such services typically provide point-of-care testing of drug samples to participants using testing strips or Fourier Transform Infrared Spectroscopy (FTIR), and some provide additional laboratory-based confirmatory testing, via various mass spectroscopy techniques, which have an inherent time delay. Confirmatory testing services typically have much higher sensitivity and specificity compared to field-based techniques and may be qualitative (describing only the presence/absence of substances contained in the drug sample) or quantitative (describing the concentration/purity of each component). Los Angeles has a long history of drug testing services (Ramesh et al., 1974; Strantz et al., 1981), although the use of these services in the fentanyl era is more recent (Shover et al., 2025).

In this analysis, we aimed to characterize samples of fentanyl submitted to a community-based drug checking program in Los Angeles, California. The primary objective was to describe the purity (concentration) of samples containing fentanyl, with secondary objectives to a) characterize the other components of these samples, and b) concentration variation across small geographic areas.

## Methods

### Sample and data collection

Using methods previously described, we leverage data from a community-based drug checking program, Drug Checking Los Angeles (Appley et al., 2023; Friedman et al., 2025; Shover et al., 2025; Sisco et al., 2017). Samples were submitted anonymously by community members for testing at 4 sites throughout Los Angeles County (located in Hollywood, East, Downtown,and South Los Angeles) between Q1 2023 and Q2 2025 (samples with quantification results available were collected between September 2023 and April 2025). Individuals providing drug samples for testing were primarily people who use drugs, and services were targeted towards this population, although no formal exclusion criteria were used to screen out other individuals wishing to use services. Participants were invited to complete an anonymous, voluntary survey that asked what they expected the drug to be, the neighborhood where they obtained it, and other characteristics of the sample. Efforts were designed to focus sample collection on retail-level drug presentations rather than those that may be considered wholesale.

Initial field testing was performed at the community sites with Fourier transform infrared (FTIR) spectrometer and immunoassay test strips, except when participants requested mail-in testing only. Laboratory testing was performed by the National Institute of Standards and Technology (NIST). All sample underwent qualitative analysis (identifying presence but not amount of over 1300 different compounds), performed with direct analysis in real-time mass spectrometry (DART-MS) (Appley et al., 2023; Russell et al., 2023; Sisco et al., 2017). A subset of these samples were subject to quantitative testing using liquid chromatography mass spectrometry (LCMS) (Shover et al., 2025). Study protocols were approved by the UCLA IRB (IRB-22-0760 and IRB-22-1273).

### Sample selection and preparation for LCMS

Samples sent for quantitative testing were scooped with a 5 mg microscoop into amber glass vials pre-filled with acetyl nitrile. Quantitative testing began in September 2023, and selection was also based on expected contents, field-test results, and availability of sample and vials (e.g. in instances where the field team had fewer vials than samples, samples provided after depletion of vials were not able to be quantified). Samples were not weighed at collection but were weighed during LCMS analysis, with that measurement used in calculating percent mass of each quantified substance in a sample. Therefore, the 5 mg sample size is an estimate reflecting the size of the scoop, but under-filled scoops were able to be analyzed per NIST protocols. All samples expected to be fentanyl, as well as those that tested positive on a fentanyl test strip or showed fentanyl on the FTIR, were sent for quantification provided there was sufficient sample to scoop and vials were available. Other samples that underwent quantification included those containing other compounds on the current quantitation panel, whether based on reported expected contents or field tests. Per protocol, samples that were expected to be a substance not included in the quantitation panel (e.g., benzodiazepines, phencyclidine, 3,4-Methylenedioxymethamphetamine (MDMA)) were only quantified if field tests showed evidence of unexpected compounds (e.g., fentanyl, methamphetamine).

### Case studies

Data collection also included a set of 3 case studies. Each represented an instance during which a number of samples were collected in Downtown Los Angeles in close time and geographic proximity. The three sessions occurred in Q3 2024, each two to four hours long, with samples obtained from the approximately one-quarter square mile of area surrounding a temporary testing site. This site was chosen given a higher density of open-air fentanyl and sales in this area, facilitating a larger number of sample collection from distinct individuals in a short period of time. The goal of this exercise and sampling strategy was to mimic the distribution of potency that the average purchaser of illicit fentanyl might experience if seeking a purchase in an area with a high density of options.

### Quantitative testing

For every sample, the quantitation panel included: fentanyl, fluorofentanyl, two fentanyl precursors (4-ANPP, phenylethyl 4-ANPP), heroin, cocaine, methamphetamine, xylazine, medetomidine, and tetracaine. A subset of samples underwent testing with expanded panels in use by NIST during part of the study period. Results were reported in percent mass, with 0.1 % imputed as the limit of quantitation.

This analysis includes all samples collected between September 2023 and April 2025 that underwent quantitative testing and were found to contain fentanyl or fluorofentanyl. This includes a period when people who use fentanyl reported declining quality and availability, and a documented decline in national and local overdose deaths.

Descriptive statistics were calculated overall and stratified into four selected categories of fentanyl purity: very low (<1 %), low (1–5 %), medium (5–15 %) and high (>15 %) purity fentanyl. These were chosen for ease of interpretability, and because they generally align with the 4 quartiles of fentanyl purity observed in the data (empirically observed quartiles were first quartile 1.4 %, median 5.0 %, third quartile 14.5 %). We also chose to stratify fentanyl concentration by several analytical categories chosen due to our a-priori interest and belief that they would be related to overdose risk. Expected drug was chosen as a stratifying variable given its importance if participants expect that they are consuming fentanyl vs. heroin, or non-opioid drugs, which could be related to their opioid tolerance and consumption practices (Friedman et al., 2022). Co-occurring substances was selected given the possibility that other adulterants are added to samples in order to increase their subjective effects, and subsequently provide a lower fentanyl concentration (Solimini et al., 2017). Formulation was chosen as a stratifying variable given the recent rise of counterfeit pills containing fentanyls on the West Coast, and our a priori belief that fentanyl concentration in pills would be lower than in powder samples (Friedman & Ciccarone, 2025; Palamar et al., 2022, 2024). Differences in median fentanyl concentration across levels of the aforementioned stratifying variables were assessed using Mood’s test of differences in medians. Pearson correlation coefficients and associated *p* values were calculated for the relationship between IMF purity and the concentration of 6 other quantified substances. Bivariate linear regression was used to assess for a relationship between date as a continuous predictor and IMF purity as a continuous outcome variable. All analyses were performed using R Version 4.4.1.

## Results

**Fig. 1.**
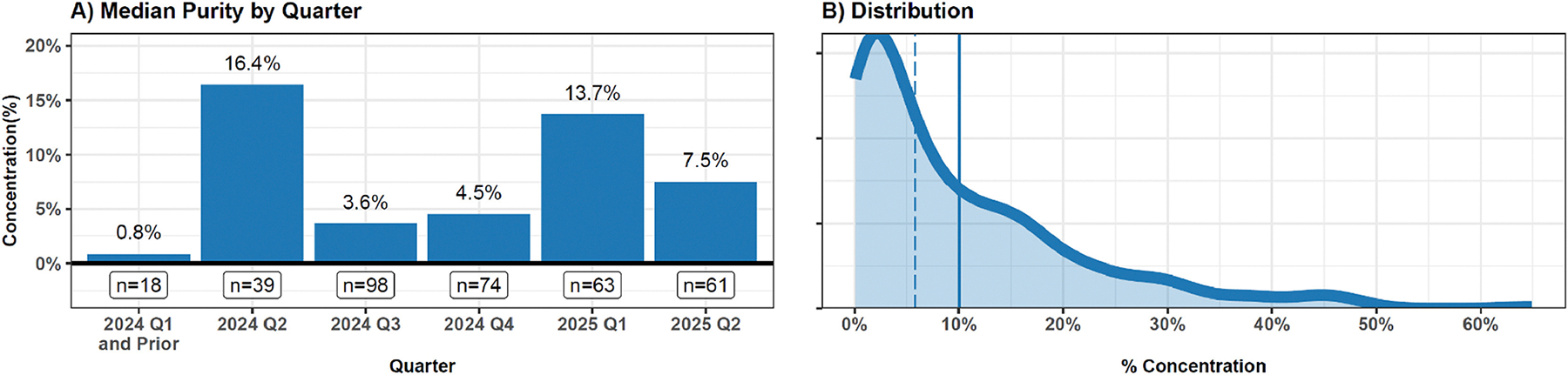
Fentanyl and fluorofentanyl concentration among fentanyl positive samples. For both plots the concentration of fentanyl and fluorofentanyl are summed and treated as a continuous variable. A) The time trend of average fentanyl and fluorofentanyl concentration is shown by quarter-year through 2025 Q2. Values prior to 2024 Q1 are grouped with 2024 Q1 due to small numbers. Sample size is shown under each bar. B) The overall distribution of fentanyl concentration (across all time points) is visualized using a kernel density estimator. The mean and median concentration are shown with a solid and dashed line, respectively.

In total, *n* = 1763 samples collected between Q1 2023 and Q2 2025 were analyzed. Of these, samples collected after September 2023, and for which at least approximately 5 mg of sample was available, were also sent for LC/MS testing. A total of *n* = 353 of these samples had quantified results available for either fentanyl (*n* = 350) or fluorofentanyl (*n* = 68), and were therefore included in this study. Only *n* = 3 samples contained fluorofentanyl but did not contain fentanyl. Fentanyl percent by mass (purity) ranged from 0.1 % to 64.9 % with median of 5.0 % and a mean of 9.6 % (standard deviation 11.0 %). Fluorofentanyl purity ranged from 0.06 % to 28.9 % with a median of 0.43 % and a mean of 3.0 % (standard deviation 5.9 %). The overall percent mass of the sum of fentanyl and fluorofentanyl (referred to as ‘fentanyl purity’ moving forward) had a median of 5.8 % and a mean 10.0 % (standard deviation 11.1 %), range 0.1 % to 64.9 %. (Table 1). Overall, a right skewed—and highly variable—distribution of fentanyl purity was noted (Fig. 1). A total of 17.0 % of samples (*n* = 60) had very low fentanyl purity (under 1 %), 30.0 % (*n* = 106) had low purity (between 1–5 %), 28.0 % (*n* = 99) had medium purity (between 5–15 %), and 24.9 % (*n* = 88) had high purity (above 15 %).

The median purity varied from quarter to quarter, with a maximum median observed in 2024 Q2 (16.4 %) and minimum observed in 2024 Q1 (0.8 %) (Fig. 1). Linear regression results indicated that there was not a statistically significant, consistent positive or negative time trend (*p* = 0.681).

Among *n* = 353 quantified fentanyl samples, the survey describing which drug participants expected their sample to represent was complete for *n* = 350 samples; *n* = 3 individuals declined this survey. Most samples (*n* = 308) were expected to be fentanyl. These samples had higher median fentanyl purity (7.0 %) compared to those expected to be heroin (*n* = 24, median purity=1.4 %) or other drugs (*p* < 0.001 (Table 1). Fentanyl concentration was particularly low among samples expected to be prescription opioids (*n* = 2, median purity 0.4 %), cocaine (*n* = 2, 0.2 %) and benzodiazepines (*n* = 1, 0.1 %). Of the *n* = 353 quantified samples that contained fentanyl/fluorofentanyl, *n* = 318 were powder, *n* = 22 were tar, and *n* = 11 were pills.(*n* = 10 expected to be opioids, *n* = 1 expected to be a benzodiazepine). Powder samples had higher median fentanyl purity (6.9 %) compared to pills (0.7 %) or tar (1.4 %) [*p* < 0.001]. Of the *n* = 308 expected-fentanyl samples, 42.5 % (*n* = 131) had an IMF purity of <5 %, while 17.5 % (*n* = 54) had purity over 20 %.

**Fig. 2.**
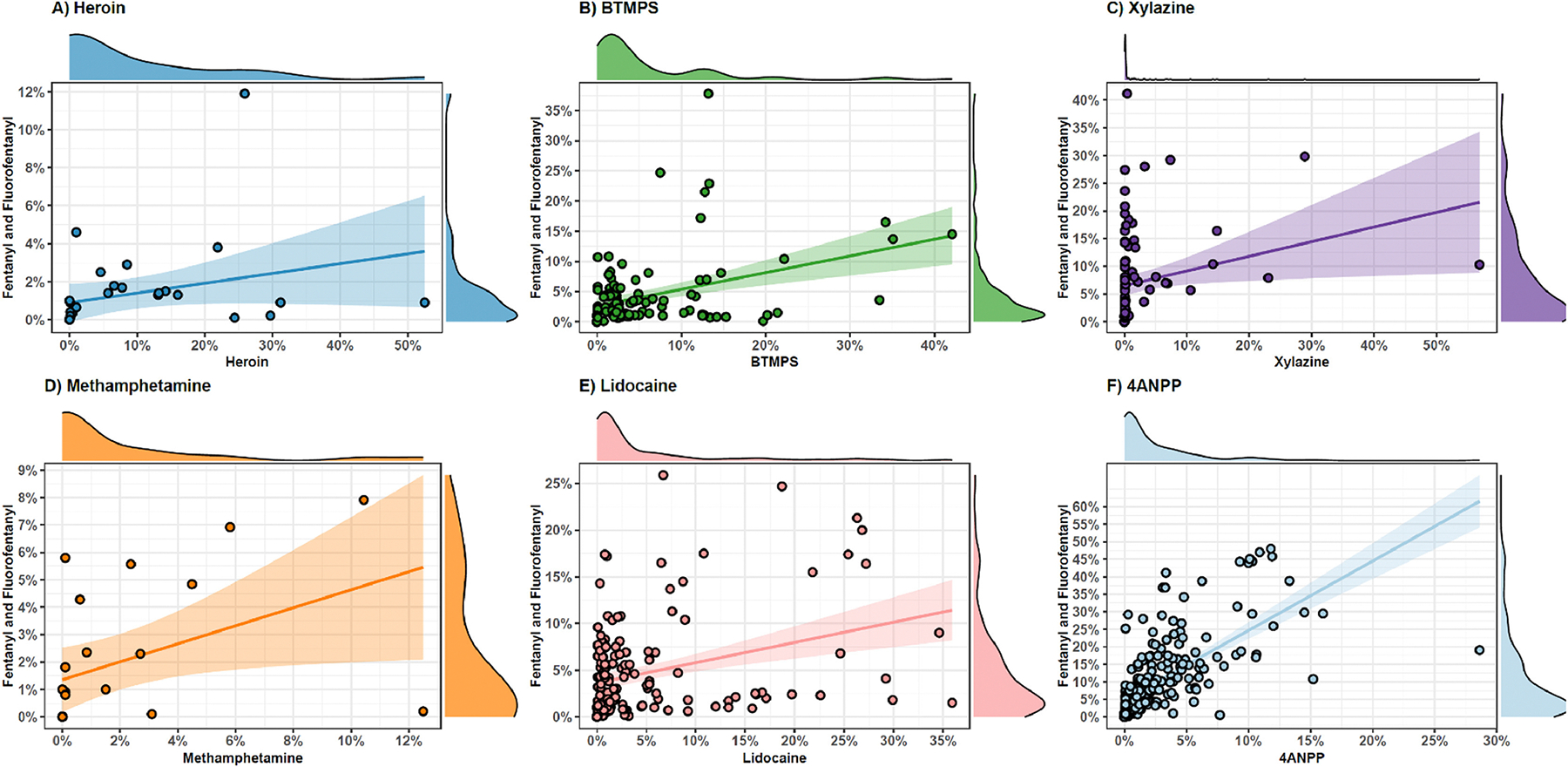
Concentration of fentanyl and fluorofentanyl compared to concentration of 6 other commonly co-detected substances For all plots the concentration of fentanyl and fluorofentanyl are summed, treated as a continuous variable, and plotted against the concentration of 6 other commonly co-detected substances. For each, a linear relationship is graphed using OLS regression, and a 95 % confidence interval of the relationship is shown. For each bivariate relationship, the distribution of both variables (fentanyl+fluorofentanyl, and the other substance shown) is visualized using a kernal density plot on each margin.

Although fentanyl purity was generally higher among samples that did not contain other co-detected substances, when looking only among the subset with quantified purity values for both fentanyl and other drugs, there was generally a positive relationship between fentanyl purity and the purity of other quantified substances (Fig. 2). All 6 substances that were quantified among a large enough sample for assessment (heroin, BTMPS, xylazine, methamphetamine, lidocaine) showed highly skewed distributions, with positive correlations to fentanyl purity (as evidenced by positive correlation coefficients). Nevertheless, only 4 of the 6 had statistically significant positive correlations with IMF purity at the alpha = 0.05 level, including BTMPS (*r* = 0.301, *p* = 0.002), xylazine (*r* = 0.209, *p* = 0.050), lidocaine (*r* = 0.333, *p*≤.001), and 4ANPP (*r* = 0.650, *p*≤.001). The correlations between IMF purity and the concentration of heroin (*r* = 0.194, *p* = 0.352) and methamphetamine (*r* = 0.327, *p* = 0.200) were not significant.

**Fig. 3.**
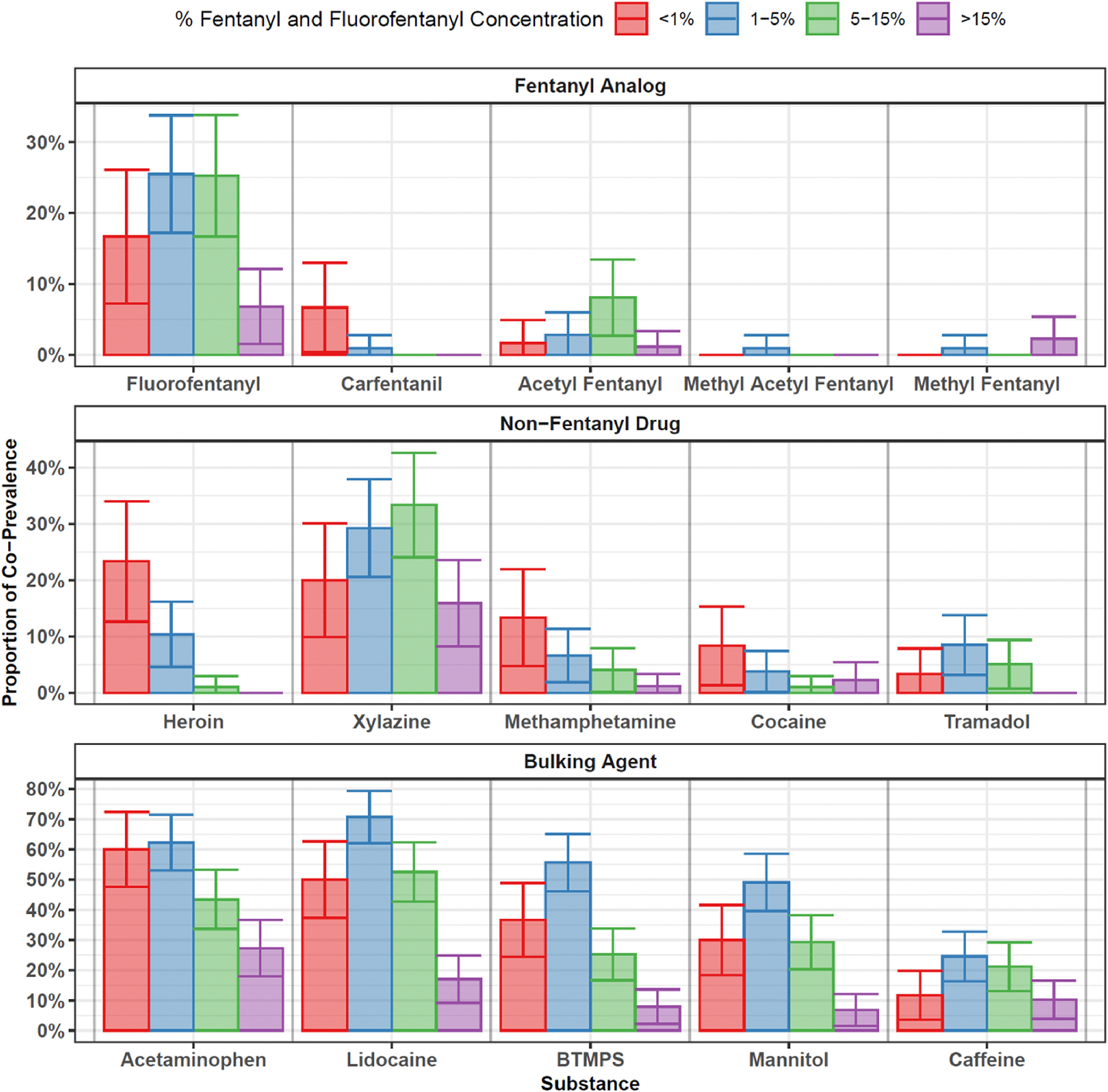
Co-prevalence of selected substances by fentanyl and fluorofentanyl concentration category. The concentration of fentanyl and fluorofentanyl is summed and treated as a categorical variable with four levels. The co-prevalence (from 0–100 %) is shown for a number of selected substances (according to present/absent status from DART-MS) separate for each categories of fentanyl concentration. The four categories roughly correspond to quartiles of fentanyl + fluorofentanyl concentration. 95 % confidence intervals around each percentage are shown with vertical bars.

Fentanyl concentration varied by presence of other co-detected substances (Table 1). Samples that were heroin-positive tended to have a lower median combined IMF purity (0.9 %) compared to heroin negative samples (6.9 %) [*p* < 0.001]. A similar trend was seen for cocaine positive vs negative samples (2.1 % vs 6.1 %; *p* = 0.041, methamphetamine positive vs negative (1.9 % vs 6.5 %; *p* = 0.003), lidocaine positive vs negative (3.5 % vs. 11.6 %; *p* < 0.001), acetaminophen positive vs negative (3.2 % vs 9.9 %; *p* < 0.001), BTMPS positive vs negative (2.5 % vs. 9.9 %; *p* < 0.001), and mannitol positive vs negative (3.0 % vs. 9.4 %; *p* < 0.001). Across these substances, the presence of a contaminant/additive was associated with lower fentanyl concentration. Similarly, co-detected substances varied by purity category (Fig. 3).

**Fig. 4.**
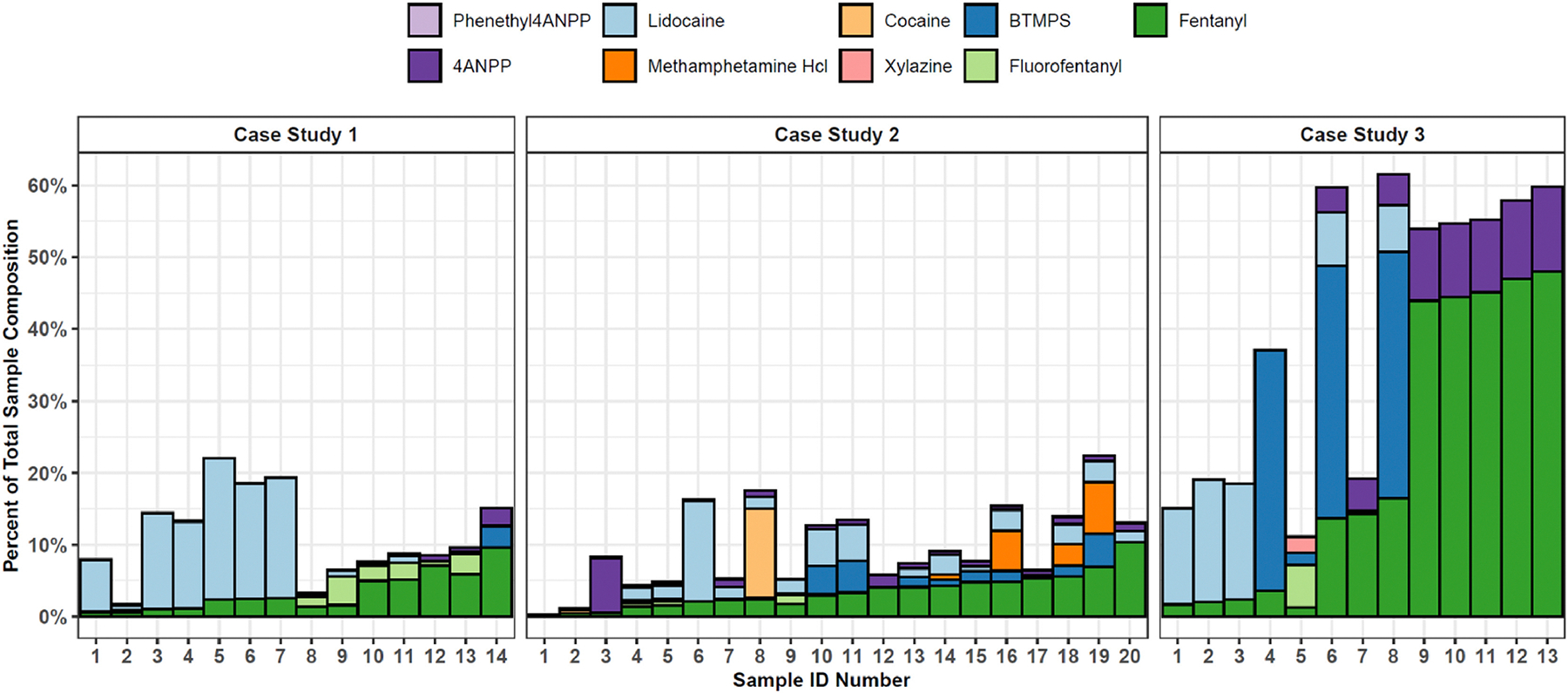
Case studies - fentanyl samples collected in close geographic and temporal proximity. This figure depicts three “case studies”. Each represents a particular day where the community-based drug checking team collected a number of samples (expected to be fentanyl) on the same day in a small geographic area. For instance, in case study 3, the 13 samples shown above were collected on a single day over the course of three hours from a geographic region in Downtown Los Angeles of approximately 10 square blocks. All samples were powder sold as fentanyl. The *x* axis shows the sample number. Each column represents one sample. The *y* axis shows the percentage of the total sample represented by each substance quantified.

In an exploratory set of 3 case studies, each representing samples collected in a single day, from a small geographic area, we observed very high variability in fentanyl concentration and other additives (Fig. 4). For instance, among the *n* = 13 total samples included in case study 3, *n* = 5 had a combined fentanyl concentration of about 50 %, *n* = 4 had a combined fentanyl concentration of about 10–20 %, and *n* = 4 had a concentration of <5 %. *N* = 3 samples had a considerable proportion of BTMPS. *N* = 5 had lidocaine. *N* = 1 sample had the majority of its combined fentanyl concentration represented by fluorofentanyl. During the other two case studies, overall concentrations were lower, without no samples above 10 % identified. During case study 2, although all samples were expected to contain fentanyl, and did contain fentanyl, *n* = 1 also contained cocaine, and *n* = 4 contained methamphetamine. This illustrates the chaotic and unpredictable nature of the illicit drug supply in Los Angeles; even in the same microneighborhood, on the same day, the fentanyl supply may, or may not, vary wildly in concentration and additives.

## Discussion

In a study of drug product samples submitted for quantitative testing at a community-based testing program in Los Angeles County, we found a large range of variability in fentanyl purity. Nearly half of the tested samples contained <5 % fentanyl by mass, while about a quarter had over 15 %, reaching as high as 65 %. In subsets of samples intentionally collected in small geographic and temporal proximity, the highest purity of a white powder sold as fentanyl exceeded the lowest by 24-fold. This variation highlights how overdose risk could change from batch to batch. To our knowledge, detailed quantitative testing of consumer-level samples of fentanyl has previously been limited. As such, these data represent a rarely seen look into what specifically is in drugs purchased as fentanyl, in a sample from the West Coast of the United States.

Generally, lower purity samples were more likely to contain compounds typically viewed as adulterants that may plausibly have clinical health effects, such as local anesthetics, acetaminophen, and the UV stabilizer BTMPS. Although samples without additives tended to have more fentanyl compared to samples with other additives, among the subset of samples with fentanyl and other quantified substances, positive relationships were seen between the purity of fentanyl and purity of other substances. This may reflect two competing phenomena: a) the presence of additives may be an alternative strategy to bolster the perceived intensity of IMF effects compared to higher IMF purity. Therefore, on average, samples with only IMF and no additives will be stronger, and also b) after subsetting to only those samples that do have both IMF and additives that can be quantified, the overall relationship is positive reflecting a correlation in the overall degree to which IMF has been diluted with inert filler components not quantified here.

The range of IMF concentration highlights how overdose risk may differ tremendously between batches obtained in very close geographic proximity. While lower purity IMF may carry reduced risk of fatal overdose compared to higher purity fentanyl, the presence of adulterants that could have additional clinical effects presents avenues for further inquiry.

The results underscore the need for expansion of quantitative drug testing in settings with IMF to understand what consumers are using. This strategy may be particularly impactful as a tool to guide preparation strategies in settings with emerging or pre-emerging IMF markets globally.

Testing for fentanyl using immunoassay test strips has been widely practiced as a harm reduction strategy, but these tests indicate only the presence and not the amount of fentanyl. As a population-level public health strategy, fentanyl test strips can sound the alarm that fentanyl has arrived in a drug market, but quantitative testing can better characterize the extent to which it is an adulterant versus a main product or “drug of choice.” Quantitative community-based drug checking may also represent an important community-led surveillance tool to improve public health. Moreover, such initiatives offer opportunities to engage with people who use drugs about various aspects of the illicit market to better characterize price, emerging substances, and other qualitative phenomena.

Further, the limited results shown here indicate that there are differences in purity by drug formulation. Although tablets or tar containing fentanyl were fewer in number, the overall range of fentanyl purity was considerably narrower than powder samples. This is likely to result in different overdose risk profiles for those that consume different formulations of drugs. Tablets and tars may be harder to tamper with, even if illegally manufactured and distributed, ensuring greater dosing consistency over powder formulations. This is something that future research and policy responses might consider as markets continue to evolve in terms of products and formulations on offer.

Additionally, it is important to note that although fentanyl purity in drug samples is likely to be an important driver of overdose risk, it is only one such driver that is likely shaping the nature of the overdose crisis. Other important factors include individual tolerance, route of administration, and the presence of other compounds, especially other sedatives that have a synergistic effect with opioids.

### Limitations

While the inclusion of quantitative testing is novel, the fact that samples came from only one metropolitan area and are inherently a convenience sample (i.e., what people choose to and are able to bring for drug checking) may limit the generalizability even within that metropolitan area. Though all samples were collected in Los Angeles County, some participants brought samples they had originally obtained from other locations. This is less of a concern in the case studies when all samples were purchased in the immediate area, but in the ongoing drug checking program there are instances of people traveling from other counties to access the service. The samples were mostly powder rather than pills, and as such the findings likely do not capture the scope of pressed pills available on the illicit market. The community-based drug checking program that generated these data sought to provide services to consumers of substances who purchase them on the retail illicit drug market. However, staff do not routinely ask participants if they are vendors, who may be bringing samples from their wholesale drug supply. Therefore, although we suspect most samples represent consumer-level purity, we cannot rule out that some fraction of samples represent wholesale products. Furthermore, we did not ask participants about the initial purchase weight that each sample was derived from, and we collect a standardized quantity (~5 mg scoop) for laboratory analysis, therefore we can only comment on purity, not sample weight.

In terms of testing methods, for most of the study period only two fentanyl analogues were quantified (fentanyl and fluorofentanyl). While these did represent the most common analogues, it is possible that not quantifying other analogues lead to an underestimate of effective fentanyl purity in these samples. However, the impact of this on the present analysis is likely small, given that only 2 % of all samples had a fentanyl analog other than fentanyl or fluorofentanyl. It is also the case that different analogues have different strengths, so adding together fentanyl and fluorofentanyl may not reflect the total potency of the fentanyls in the drug, particularly since different fluorofentanyl isomers have different potency (Canfield et al., 2025). Although some studies have estimated fluorofentanyl to have a potency of 1.3 times that of fentanyl, other studies have found them to be equipotent (Canfield et al., 2025). Given that fluorofentanyl was far less common than fentanyl in this study, and most studies find them to be on the same order of magnitude of potency, the decision to combine their concentrations for this study likely did not have a large effect on the study results.

Of note, this study provides a description of fentanyl potency and several related factors. However future research can more formally explore predictors of fentanyl potency, and assess to which degree they are consistent between distinct geographies.

In conclusion, we found extreme variability in the purity of fentanyl in consumer-level samples, often in samples obtained within the same small geographic area on the same day. This variation may partially explain overdose risk among people with opioid tolerance, and may point the way for community-led drug supply surveillance efforts to understand risk and harm for people who use opioids. Findings about differing co-detected compounds may suggest avenues to study different synthesis methods and manufacturing decisions (i.e. using different diluents to bulk up a sample). Quantitative testing of illicitly manufactured drugs is poised to make unique contributions in understanding and responding to changes in the illicit drug supply that ultimately impact public health.

## Figures and Tables

**Table 1.** Fentanyl and fluorofentanyl concentration by expected substance, sample form, and co-prevalent substances.

Grouping Variable	Purity Median - Mean (SD; Range)	P Value	
Overall	Overall (*n* = 353)	5.8–10.0 % (11.1 %; 0.1–64.9 %)	–
Expected Substances	Fentanyl (*n* = 308)	7.0–10.9 % (11.4 %; 0.1–64.9 %)	**<0.001** *
Heroin (*n* = 24)	1.4–2.7 % (3.5 %; 0.1–11.9 %)		
Unspecified Opioids (*n* = 6)	0.7–0.6 % (0.4 %; 0.1–1.2 %)		
Xylazine+Fentanyl (*n* = 4)	11.2–13.2 % (7.0 %; 7.7–22.6 %)		
Declined (*n* = 3)	18.4–13.1 % (11.3 %; 0.2–20.8 %)		
Unknown (*n* = 3)	3.9–8.4 % (9.0 %; 2.5–18.7 %)		
Prescription Opioids (*n* = 2)	0.4–0.4 % (0.2 %; 0.3–0.5 %)		
Cocaine (*n* = 2)	0.2–0.2 % (0.1 %; 0.1–0.2 %)		
Benzodiazepines (*n* = 1)	0.1–0.1 % (NA %; 0.1–0.1 %)		
Sample Form	Powder (*n* = 318)	6.9–10.8 % (11.3 %; 0.1–64.9 %)	**<0.001** *
Tar (*n* = 22)	1.4–3.2 % (4.5 %; 0.1–18.4 %)		
Pill (real + pressie) (*n* = 11)	0.7–1.4 % (2.2 %; 0.1–7.5 %)		
Residue (*n* = 1)	7.9–7.9 % (NA %; 7.9–7.9 %)		
Other (*n* = 1)	9.9–9.9 % (NA %; 9.9–9.9 %)		
**Co-Prevalent Substances**			
**Fentanyl Analogs**			
Fentanyl	Present (*n* = 350)	5.8–10.0 % (11.1 %; 0.1–64.9 %)	1
Absent (*n* = 3)	1.5–6.0 % (9.0 %; 0.1–16.4 %)		
Fluorofentanyl	Absent (*n* = 285)	6.7–10.9 % (11.7 %; 0.1–64.9 %)	0.0839
Present (*n* = 68)	3.6–6.4 % (7.1 %; 0.1–29.8 %)		
Acetyl Fentanyl	Absent (*n* = 340)	5.6–10.1 % (11.3 %; 0.1–64.9 %)	0.2539
Present (*n* = 13)	11.0–8.9 % (5.2 %; 0.9–15.3 %)		
Carfentanil	Absent (*n* = 348)	5.9–10.1 % (11.1 %; 0.1–64.9 %)	0.07261
Present (*n* = 5)	0.1–0.6 % (0.9 %; 0.1–2.2 %)		
Methyl Acetyl Fentanyl	Absent (*n* = 352)	5.8–10.0 % (11.1 %; 0.1–64.9 %)	1
Present (*n* = 1)	3.9–3.9 % (NA %; 3.9–3.9 %)		
Methyl Fentanyl	Absent (*n* = 350)	5.8–10.0 % (11.1 %; 0.1–64.9 %)	0.9961
Present (*n* = 3)	16.4–12.5 % (9.6 %; 1.5–19.5 %)		
**Non-Fentanyl Drugs**			
Tramadol	Absent (*n* = 337)	6.0–10.3 % (11.2 %; 0.1–64.9 %)	0.2049
Present (*n* = 16)	2.9–3.7 % (3.0 %; 0.1–9.0 %)		
Xylazine	Absent (*n* = 263)	5.8–10.7 % (11.9 %; 0.1–64.9 %)	0.9275
Present (*n* = 90)	5.8–8.0 % (8.1 %; 0.1–41.1 %)		
Cocaine	Absent (*n* = 341)	6.1–10.2 % (11.1 %; 0.1–64.9 %)	**0.041** *
Present (*n* = 12)	2.1–5.7 % (9.1 %; 0.1–26.7 %)		
Methamphetamine	Absent (*n* = 333)	6.5–10.4 % (11.2 %; 0.1–64.9 %)	**0.003** *
Present (*n* = 20)	1.9–3.9 % (6.6 %; 0.1–29.8 %)		
Heroin	Absent (*n* = 327)	6.9–10.7 % (11.2 %; 0.1–64.9 %)	**<0.001** *
Present (*n* = 26)	0.9–1.6 % (2.4 %; 0.1–11.9 %)		
**Bulking Agents**			
Lidocaine	Absent (*n* = 181)	11.6–14.3 % (13.1 %; 0.1–64.9 %)	**<0.001** *
Present (*n* = 172)	3.5–5.5 % (5.6 %; 0.1–25.9 %)		
Acetaminophen	Absent (*n* = 184)	9.9–13.2 % (12.6 %; 0.1–64.9 %)	**<0.001** *
Present (*n* = 169)	3.2–6.5 % (7.8 %; 0.1–45.8 %)		
BTMPS	Absent (*n* = 240)	9.9–12.5 % (12.0 %; 0.1–64.9 %)	**<0.001** *
Present (*n* = 113)	2.5–4.8 % (6.0 %; 0.1–37.8 %)		
Caffeine	Absent (*n* = 290)	6.8–10.7 % (11.7 %; 0.1–64.9 %)	0.1722
Present (*n* = 63)	4.6–7.0 % (7.1 %; 0.1–29.0 %)		
Mannitol	Absent (*n* = 248)	9.4–12.2 % (12.1 %; 0.1–64.9 %)	**<0.001** *
Present (*n* = 105)	3.0–4.9 % (5.5 %; 0.1–28.0 %)		

For all groups the concentration of fentanyl and fluorofentanyl is summed and treated as a continuous variable. This is shown overall, as well as broken down by expected substances, sample form, and a number of co-prevalent substances (present/absent according to DART-MS). Co-prevalent substances are sorted by class, which is shown by color. *p* values are shown using Mood’s test of differences in medians.

* Significant at 0.05 level.
